# Effect of Anionic Surfactants on the Oil–Water–Rock Interactions by an Improved Washburn Method

**DOI:** 10.3390/molecules29122878

**Published:** 2024-06-17

**Authors:** Tie Kuang, Yubo Lan, Zhilin Yin, Xin He, Wanquan Tang, Yan Wang, Zheng Wang, Feng Yan, Lu Zhang

**Affiliations:** 1 Heilongjiang Key Laboratory of Reservoir Physics&Fluid Mechanics in Porous Medium, Daqing 163712, China; kuangtie@petrochina.com.cn (T.K.); lanyb@petrochina.com.cn (Y.L.); yinzhl@petrochina.com.cn (Z.Y.); hexin01@petrochina.com.cn (X.H.); wqtang@petrochina.com.cn (W.T.); wangyan6619@petrochina.com.cn (Y.W.); 2 Exploration and Development Research Institute of PetroChina Daqing Oilfield Co., Ltd., Daqing 163712, China; 3 College of Petroleum Engineering, China University of Petroleum (East China), Qingdao 266580, China; wangzheng19970816@163.com; 4 School of Chemistry, Tiangong University, Tianjin 300387, China; 5 Key Laboratory of Photochemical Conversion and Optoelectronic Materials, Technical Institute of Physics and Chemistry, Chinese Academy of Sciences, Beijing 100190, China

**Keywords:** Washburn, anionic surfactants, oil sand, contact angle, wettability

## Abstract

The complex and variable structure of subsurface oil reservoirs as well as the small pore throat size of reservoirs make it extremely important to investigate the effect of oil–water–rock interactions for enhancing oil recovery. In this paper, the powder wettability of oil sand with different polar solvents was investigated using the improved Washburn capillary rise method, and the surface free energy of oil sand was calculated in combination with the OWRK method. In addition, the wettability of anionic surfactants HABS and PS solutions on the surface of oil sand was determined, and it showed that their wetting rates showed different trends after CMC (critical micelle concentration). The C×cosθ value of HABS decreased significantly with increasing concentration, whereas PS showed little changes. This may be related to the aggregate structure formed by HABS on the oil sand surface. Meanwhile, the interfacial free energy between crude oil and oil sand was obtained by crude oil-to-oil sand wetting experiments, and found that the wetting rate of crude oil to oil sand was much lower than that of solvents and surfactants. In combination with the above results and the oil–water interfacial tension (IFT), the oil–water–rock three-phase contact angle and the work of adhesion between the crude oil and the solid were obtained by Young’s equation. From the three-phase contact angle results, it can be found that the contact angle values of both HABS and PS are obviously higher than that of the simulated water, and both HABS and PS have the ability to significantly reduce the work of adhesion, which shows a strong ability to strip the oil film on the surface of the solid. The research results of this paper are helpful to understand the oil displacement mechanism of chemical flooding in reservoir pores, which is of great significance for improving oil recovery.

## 1. Introduction

The development and utilization of petroleum resources has a pivotal position in the context of sustainable economic development. Low-permeability oil reservoirs in the underground are rich in reserves, but the exploitation effect is not ideal [1,2,3]. Due to the complex and variable structure of underground oil reservoirs in low-permeability reservoirs and the small size of reservoir pore throats, the rock–solution interaction has a large impact on the oil displacement efficiency. The seepage of the displacement fluid in the reservoir pore space is closely related to the interfacial interaction between the fluid and the rock, while the interfacial interaction between the crude oil and the rock directly affects the microscopic displacement efficiency. Especially for low-permeability reservoirs, the effect of pressure reduction and injection increase and the improvement of microscopic sweep efficiency can be achieved by regulating the wettability of fluid on the rock surface [4,5,6]. Therefore, the study of molecular interactions at the solid–liquid interface is an important part of the research on the mechanism of improving recovery in low-permeability reservoirs.

For the determination of surface wettability of solid particles, there are the sessile drop method, Williams plate, liquid penetration and Washburn’s capillary rise method [7]. The Washburn capillary rise method is used to determine the wettability of a powder by filling it in a sample tube and utilizing the spontaneous wetting behavior of the solution on the powder. Compared to the other methods, the Washburn method uses a tight filling of the powder. This allows for the consistency of results from one experiment to the next and also reduces the possibility of the powder collapsing during the experiment and the volatility of the solution in the air [8].

In recent years, many papers have reported the Washburn capillary rise method for the determination of surface wettability of solid particles. Klimenko et al. [9] investigated the wettability of quartz sand and limestone powder by Washburn’s method using brines with different mineralization and found that the powder contact angle varied with the change in the salinity. Yang et al. [10] determined the wettability of magnetite, zeolite, manganese sand, quartz sand and ceramic sand particles with particle sizes ranging roughly from 0.45 to 0.9 mm based on Washburn’s equation and capillary rise method with deionized water and cyclohexane as solvents. Their lipophilic-to-hydrophilic ratio (LHR) values were calculated as 1.057, 0.640, 0.736, 0.652 and 0.877, respectively. The results revealed that magnetite was the most hydrophobic particle, and zeolite was the most hydrophilic one. Xiang et al. [11] determined the wettability of cyclohexane and deionized water on vermiculite, silica nanosheets (acid-treated vermiculite) and montmorillonite, by using the Washburn capillary ascent method, according to which their LHR values were found to be greater than 1, which proved that the modified particles had some hydrophobicity. Wang et al. [12] explored the Washburn capillary rise method for determining the wettability of hydrophobized kaolinite, and the solvents were chosen as hexane, toluene, methanol, dichloromethane and hexane. It was found that the hydrophobicity of the hydrophobized kaolinite gradually increased with the gradual increase in the mass ratio of kaolinite to toluene. Kong et al. [13] investigated the effect of powder particle size on the surface wettability of clay minerals by using the Washburn capillary rise method to determine the surface wettability of deionized water, formamide and glycerol. It was found that the powder contact angle gradually decreased as the particle size of the clay minerals decreased. Jaine et al. [14] measured the powder wettability of solvent on catalyst carriers by the Washburn capillary rise method. Fifteen different solvents such as methanol, ethanol and heptane were selected as solvents, and silica, alumina, hydroxyapatite and microcrystalline cellulose powders were selected as catalyst carriers. It was found that the powder contact angles were consistently lower for silica and alumina and significantly higher for hydroxyapatite and microcrystalline cellulose.

On this basis, some literature works have obtained the wettability of different types of surfactant solutions on the surface of solid particles. Zelenev et al. [15] determined the wettability of nonionic surfactant C_12_EO_7_ on different types of oil-bearing sandstone particles and found that the increase in height of the solution in the capillary with the increase in surfactant concentration indicates a gradual increase in the ability to wet the particles. Chen et al. [16] studied the modification of diesel fuel by surfactants 2-ethylhexanol and docosyl dimethyl ammonium bromide (DDAB) by using Washburn’s dynamic capillary method. They found that the addition of surfactants increased the wetting ability of modified diesel fuel on low-order coal samples. Bi et al. [17] investigated the wettability of sodium dodecylbenzene sulfonate (SDBS) solution on hydrophobically modified silica gel powder by the Washburn method. It was found that with the increasing concentration of SDBS solution, the powder contact angle of the solution on the surface of silica gel powder showed a tendency to decrease and then increase gradually. Fu et al. [18] mixed montmorillonite clay (MMT) with three surfactants, octadecylamine (ODA), hexadecyltrimethylammonium bromide (HTAB) and benzalkonium chloride (BAC), and an in-house synthesized surfactant, vinylbenzylalkyldimethylammonium chloride (VDAC). The wettability of styrene and toluene solutions on the organoclay–surfactant mixtures was investigated by the Washburn method. It was found that the wetting slopes of ODA-MMT, HTAB-MMT and BAC-MMT increased gradually, but the slower wetting rate of VDAC-MMT resulted in a nonlinear wetting slope.

As is known, wettability controls the distribution of fluids in porous media and thus affects the multiphase flow during enhanced oil recovery. For the enhancement of crude oil recovery, most studies have been conducted to change the surface wettability of subsurface oil reservoir rocks by injecting specific types of surfactants [19,20]. However, most of the experimental studies investigate the surface wettability of particles from a macroscopic point of view, while there are fewer studies on the interaction of surfactants with subsurface oil reservoir rocks from a microscopic point of view. Therefore, in this paper, the powder wettability of oil sand was determined by combining the Washburn capillary rise method, while the surface free energy of oil sand was calculated by the OWRK method. Similarly, the powder contact angle of the anionic surfactant on the surface of the oil sand and the interfacial free energy between the oil sand and the anionic surfactant solutions were determined. Further, the powder contact angle and the interfacial free energy between the crude oil and the oil sand were investigated using the method described above. The effects of anionic surfactants on oil–water–rock interactions were investigated by combining the above experimental results and calculating the three-phase contact angles between crude oil, anionic surfactants and oil sand by Young’s equation.

## 2. Results and Discussion

### 2.1. Surface Free Energy of Oil Sand

#### 2.1.1. Wettability on Oil Sands

**Figure 1:**
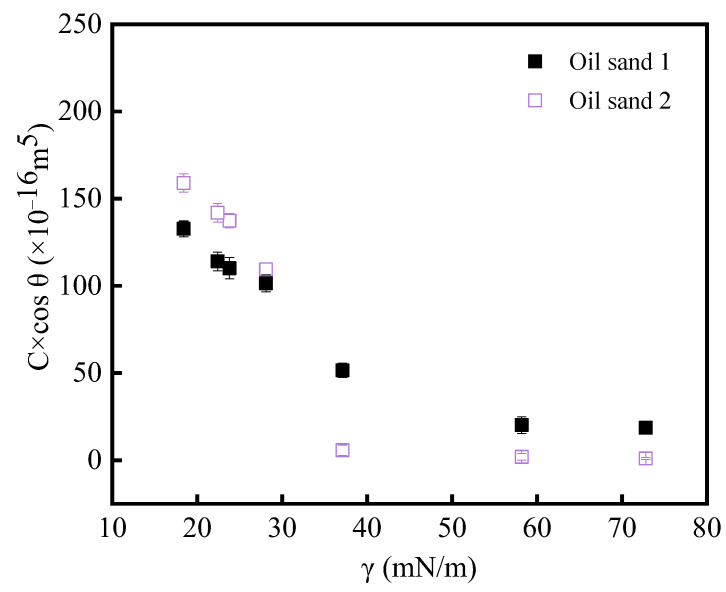
C×cosθ values of different solvents for oil sands 1 and 2.

For simplicity, the original wetting curves are not provided, and the wetting curves are similar to previous literature [21]. Figure 1 shows the C×cosθ (θ is the powder contact angle) values of oil sands 1 and 2 in water, formamide, DMF, ethanol, cyclohexane, n-decane and n-hexane, which were obtained by substituting the results of the wettability measurements of the above different solvents for oil sands 1 and 2 in Equation (4).

From Figure 1, it can be found that for oil sands with the same particle size, the wetting rate of different solvents decreases with the increase in surface tension. As the value of surface tension of solvent increases gradually, the C×cosθ value of powder shows two stages. The first stage is when the surface tension value is lower, and it can be found that with the gradual decrease in surface tension, the C×cosθ values of different solvents vary greatly and show a trend of gradual increase. The second stage is when the surface tension is greater than 40 mN/m, in which the C×cosθ values of different solvents do not vary much. The experimental results of Wang et al. [21] also found that the C×cosθ values of quartz sand with different particle sizes in the solvents of water, formamide, DMF (N,N-Dimethylformamide), ethanol, cyclohexane, n-C_10_ and n-C_6_ also showed a two-stage trend, which is similar to the experimental results for the oil sand in Figure 1.

In addition, Chang et al. [22] proposed the LHR based on the Washburn method by determining the wettability of cyclohexane and water on nut shells, manganese sand, ceramic particles, quartz sand and ceramic sand, and the wetting slopes were brought into the equations to calculate the LHR values of different solid particles, and the obtained LHR values were 66.87, 1.24, 1.22, 1.16 and 0.80. It was found that the nut shells were strongly lipophilic, while the quartz sand showed some hydrophilicity. The results of Figure 1 were substituted into the LHR equation, and the LHR values of different oil sands were calculated. It was found that the LHR of oil sand 1 and oil sand 2 were 5.46 and 28.91, respectively. Considering the measurement error, the LHR was acceptable. Through comparison, it is found that the oil sand selected in this paper has strong lipophilicity. Therefore, the results of measuring the wettability of oil sand by the Washburn method in this paper are consistent with the qualitative results in the literature.

#### 2.1.2. Powder Contact Angle of Oil Sand

Wang et al. [21] calculated the value of capillary constant C for quartz sands with different particle sizes by determining the gas–liquid–solid contact angle on quartz sand with different particle sizes of deionized water, while combining with the Washburn capillary rise method. At the same time, the capillary constant C of quartz sand was linearly fitted, and the equation obtained was C = 0.052 × d − 1.195. The particle size of the oil sand particles was 147 μm, and it was assumed that the oil sand particles were all spherical-like particles. Then, the particle size of the oil sand particles was substituted into the fitting equation of the capillary constant C of quartz sand, and the capillary constant C of the oil sand particles was obtained to be 6.45 × 10^−16^ m^5^. Substituting the capillary constant C of the oil sand particles into the value of C×cosθ, the obtained values of the powder contact angles of different solvents (pure water, formamide, DMF, ethanol, cyclohexane, n-C_10_ and n-C_6_) on the oil sand particles are shown in Table 1.

The combination of the powder contact angles of different solvents on oil sand in the above table reveals that the water contact angle values on oil sand 1 and 2 particles are roughly around 89.0°. Nikakhtari et al. [23] determined the water contact angle of oil sand to be 80–88° by the solid droplet method. Wang et al. [12] also measured the water contact angle of oil sands (about 83–89°) by the Washburn capillary rise method and obtained similar results. Meanwhile, Liu et al. [24] determined the water contact angle of oil sand particles to be 88.8 ± 0.9° based on the Washburn method. Thus, the results indicate that oil sands have high hydrophobicity and low polarity.

In addition, it can be seen from Table 1 that the powder contact angle of different solvents decreases gradually with the decrease in solvent surface tension. When the polarity of the solvent is high, the polarity difference between the solvent and the oil sand is large, and the solid–liquid interfacial free energy is high, which makes the solvent’s contact angle on the surface of the oil sand have a higher value. At the same time, due to the lower polarity of the oil sand, the gas–solid interface free energy of the system is lower. As a result, the rate of solvent wetting the oil sand is lower, and the C×cosθ value is lower. When the polarity of the solvent is low, the polarity difference between the solvent and the oil sand is not much. As a result, the value of the powder contact angle of the oil sand decreases with the decrease in solvent polarity.

#### 2.1.3. Calculation of Surface Free Energy of Oil Sand

The cosθ values are substituted into Equation (7), and at the same time, according to the polar component of different solvents $\gamma_{l}^{p}$ and nonpolar component $\gamma_{l}^{d}$, and then the obtained $\gamma_{s}^{d}$ and $\gamma_{s}^{p}$ are added together to obtain $\gamma_{s}$ as the surface free energy of the oil sand particles. The results of the calculations are shown in Table 2. The surface free energies of oil sands 1 and 2 were calculated to be 24.52 and 23.80 mJ/m^2^, respectively. From the above, it can be seen that the oil sands have high hydrophobicity, and the values of $\gamma_{s}^{d}$ are obviously higher than those of $\gamma_{s}^{p}$. 

### 2.2. Free Energy of the Oil Sand–Surfactant Solution Interface

#### 2.2.1. Surface Tension of Surfactant Solutions

**Figure 2:**
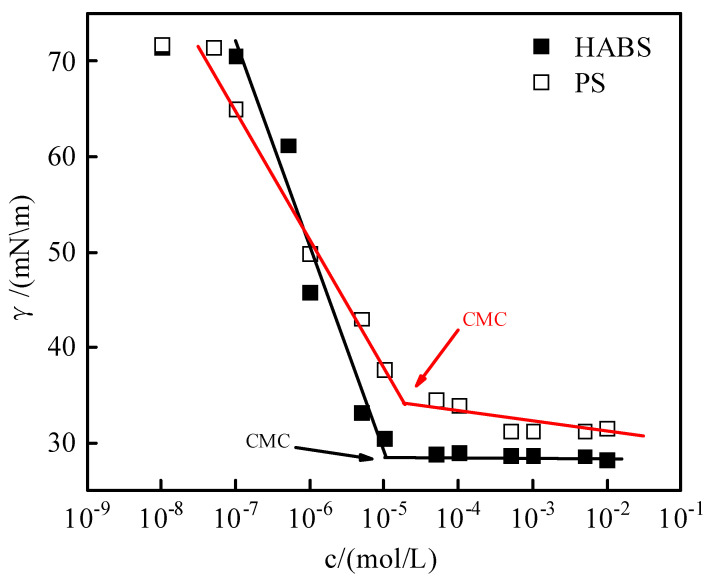
Concentration dependence curves of surface tension of surfactants HABS and PS.

Surface tension was measured for HABS (heavy alkyl benzene sulfonate) and PS (petroleum sulfonate) solutions as a function of concentration, as shown in Figure 2.

As shown in Figure 2, the surface tension of anionic surfactants HABS and PS decreased linearly with concentration and then reached a plateau. With the increase in surfactant concentration, more and more surfactant molecules are adsorbed to the gas–liquid interface, the surface tension gradually decreases, and the surfactant molecules form micelles in solution when their concentration reaches the critical micelle concentration (CMC) [25]. At this time, the surface tension reached a plateau value above the CMC, which was 1.01 × 10^−5^ mol·L^−1^ and 2.85 × 10^−5^ mol·L^−1^ for HABS and PS, respectively. The γ_cmc_ values were also obtained for the surfactants HABS and PS, which were 29.09 mN·m^−1^ and 34.19 mN·m^−1^, respectively. In addition, the surface tension of the simulated water value was 71.38 mN·m^−1^.

#### 2.2.2. Powder Contact Angle of Surfactant Solution on Oil Sands

**Figure 3:**
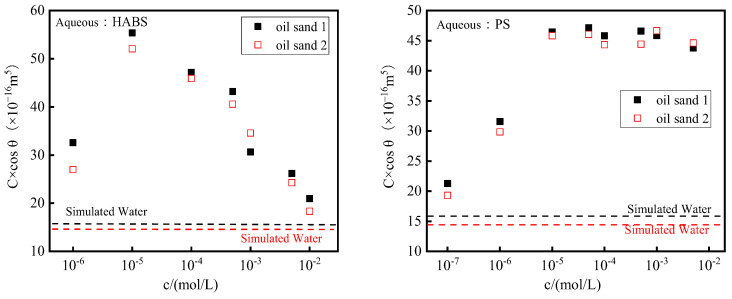
C×cosθ values of HABS and PS solutions on oil sands.

The C×cosθ values of HABS and PS solutions on oil sands 1 and 2 are plotted as a function of concentration, as shown in Figure 3. From the figure, it can be found that the C×cosθ values of HABS on oil sand show a tendency of increasing and then decreasing with the gradual increase in surfactant concentration after CMC. Differently, the C×cosθ value of PS on oil sand showed a trend of increasing to the plateau value at CMC. In addition, the C×cosθ of the surfactant solution was always higher than that of the simulated water.

From the above, it can be seen that the polarity of oil sand is lower, and the gas–solid interfacial free energy is lower. When the concentration of surfactant is low, the surface tension is higher, the capillary force is lower, and the wetting rate is lower. The team of Zelenev [15] investigated the wetting experiments of C_12_EO surfactant solutions on oil sand by the Washburn technique and found that the height of the solution wetting the powder increased gradually with the increase in surfactant concentration. They concluded that the hydrophobic alkyl of C_12_EO adsorbs on the oil sand surface through hydrophobic interactions, while the hydrophilic portion is directed toward the aqueous, causing the hydrophilicity of the oil sand surface to gradually increase. Similarly, the anionic surfactants HABS and PS can also adsorb on the oil sand surface through hydrophobic interactions, and hydrophilic sulfonic acid groups are directed away from the surface of the oil sand to increase the hydrophilicity of the oil sand gradually [26]. This leads to an increase in the capillary force on the droplets, an increase in the solution wetting rate, and an increase in the C×cosθ value. Moreover, the decrease in surface tension also benefits the increase in C×cosθ value before CMC.

Notably, for the HABS solution, when the concentration is greater than CMC, the C×cosθ value decreases significantly with increasing concentration, implying that the high concentration of HABS causes a decrease in the hydrophilicity of the oil sand surface, which may be because, at this time, the HABS molecules form aggregates on the surface of the oil sand. While PS molecules are larger and do not easily form solid surface aggregates, therefore, the value of C×cosθ was unchanged after CMC.

Substituting the capillary constant C of the oil sand particles into the above C×cosθ value, the results obtained are the powder contact angles of different concentrations of surfactant HABS and PS solutions on oil sand 1 and oil sand 2. The experimental results are shown in Table 3.

From Table 3, it can be found that the powder contact angles of HABS and PS solutions on oil sand do not change much on the whole, which is on the one hand because the anionic surfactants adsorb to the oil sand surface through hydrophobic interaction, with low adsorption amount and weak hydrophilic modification of oil sand, and on the other hand, since the oil sand is a low-energy surface, the contact angle of the surfactant solution is close to 90°, and the influence of the surface tension component on the adherence tension is small. Therefore, the overall effect of anionic surfactant on the contact angle of powder is small.

#### 2.2.3. Calculation of Free Energy at the Oil Sand–Surfactant Solution Interface

The solid–liquid interfacial free energies of HABS and PS solutions on oil sands 1 and 2 were calculated by bringing the powder contact angles, surface tension values and the surface free energies into the Young’s equation, and the results are shown in Table 4.

From the table, it can be found that for oil sand, the interfacial free energy between HABS and oil sand shows a trend of slightly decreasing first with the surfactant concentration, and then slightly increasing after the CMC, while PS slightly decreases to the plateau value in general. However, the overall changes were not significant. As mentioned before, the anionic surfactant is adsorbed on the surface of oil sand through hydrophobicity, the adsorption amount is low, and its hydrophilic modification ability to oil sand is weak. This leads to little change in the interfacial free energy between the anionic surfactant solutions and the oil sand.

### 2.3. Oil–Water–Oil–Sand Contact Angle

#### 2.3.1. Interfacial Free Energy of Crude Oil–Oil Sand

The wettability of Daqing crude oil to oil sands 1 and 2 was determined by the Washburn capillary rise method at the oil reservoir temperature (45 °C), and the C×cosθ values of oil sands 1 and 2 in crude oil were obtained by calculation. The surface tension of the crude oil at 45 °C was measured to be 29.59 mN/m. In the same way as that of the oil sand and surfactants, the powder contact angle of the crude oil on the oil sand was calculated by bringing the capillary constant C of the oil sand particles into the C×cosθ value. According to the powder contact angle of crude oil on oil sand and the surface free energy of oil sand combined with the surface tension of crude oil, the solid–oil interfacial free energy of oil sand–crude oil can be obtained from the Yang equation, and the results are shown in Table 5.

As we know, crude oil is mainly composed of alkane molecules, which are nonpolar substances [27]. As can be seen from the previous section, oil sands have lower surface free energy and lower polarity, so the liquid–solid interface free energy of the system is lower. Also from the table, it can be found that the C×cosθ value of oil sand in crude oil is relatively low compared to the C×cosθ value of oil sand in surfactants.

In addition, the presence of some polar substances in crude oil affects the original density and viscosity [28]. Therefore, the wetting rate of crude oil infiltrating oil sand is low. The C×cosθ values of the oil sand are also low, and the calculated powder contact angle of the crude oil on the surfaces of oil sands 1 and 2 is about 88°.

Meanwhile, PTFE is a highly nonpolar material with a surface free energy of 22.34 mJ/m^2^; whereas, the surface free energy of oil sands is between 23 and 25 mJ/m^2^, so oil sands are also highly nonpolar surfaces [29]. In addition, n-decane is a nonpolar liquid with a surface tension of 23.8 mN/m; the surface tension of crude oil is 29.59 mN/m (45 °C), which makes crude oil a weakly polar liquid. Therefore, there is a certain polarity difference between crude oil and oil sands, and the larger the polarity difference, the larger the interfacial free energy. Based on this, it is reasonable that the solid–oil interfacial free energy of oil sands and crude oil is about 23 mJ/m^2^.

#### 2.3.2. Oil–Water Interfacial Tension

**Figure 4:**
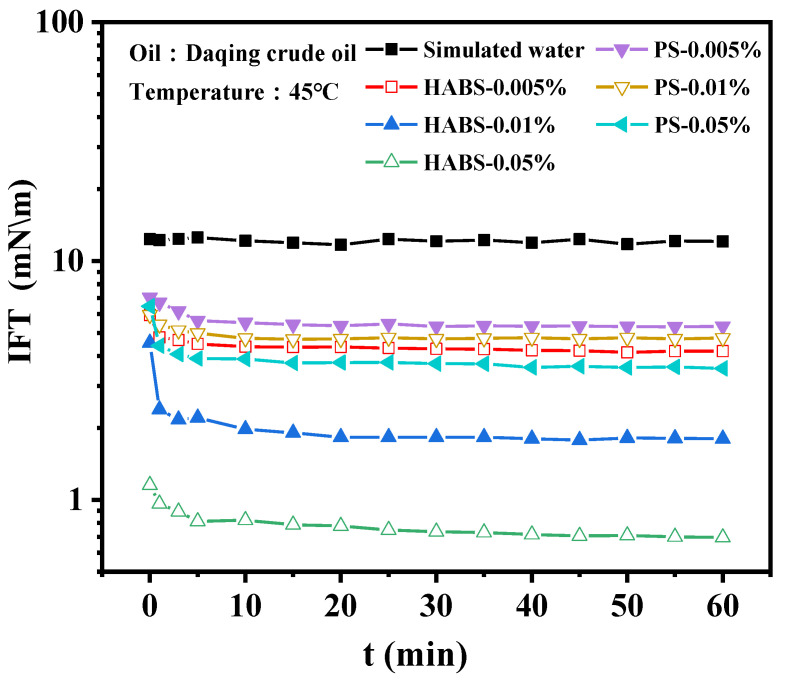
Dynamic graphs and steady-state values of IFT on Daqing crude oil at different concentrations of HABS and PS.

The dynamic graphs of interfacial tension (IFT) of HABS and PS solutions with different concentrations against Daqing crude oil are shown in Figure 4.

From Figure 4, it can be found that the dynamic IFT curves are all “L”-shaped, the IFT decreases gradually until it reaches equilibrium with time [30]. The surfactant molecules migrate toward the oil–water interface and gradually replace the solvent molecules at the interface. Hydrophilic groups give the molecules a tendency to enter water, while hydrophobic groups try to prevent their solubilization in water [31]. The balance of these two tendencies leads to the enrichment of surfactant molecules at the oil–water interface. Meanwhile, the IFT of HABS and PS showed a gradual decrease with the increase in surfactant concentration. The IFT of HABS reaches 10^−1^ mN/m order of magnitude, while PS is one order of magnitude higher than HABS.

#### 2.3.3. Oil–Water–Oil Sand Contact Angle

According to Young’s equation, it can be seen that in the oil–water–solid system, the role of surfactants is mainly to change the wettability of the rock surface, which affects the adsorption of crude oil on the rock surface and promotes the stripping of the oil film. The three-phase contact angles between crude oil, water and oil sands were obtained by bringing the IFT, liquid–solid interfacial free energy and solid–oil interfacial free energy of HABS and PS solutions with different mass concentrations into Young’s equation and Equation (11). Table 6 and Table 7 show the three-phase contact angles and the values of adhesion work for HABS and PS, respectively.

From Table 6 and Table 7, it can be found that the three-phase contact angles of oil–water–oil sand for simulated water are 96.5° and 93.9°, respectively. The contact angle values of different concentrations of HABS and PS were higher than that of simulated water, and with increasing concentration, the contact angle values of HABS obviously increased, and those of PS slightly increased. This is due to the adsorption of anionic surfactant at the water–oil–sand interfaces through the action of hydrophobic properties, which reduces the free energy of the solid–liquid interface; at the same time, the surfactant molecules are enriched at the oil–water interface, which results in a significant decrease in the interfacial tension between oil and water. Then, both factors are favorable to its further elevation when the contact angle is higher than 90°. In addition, there is also a kinetic mechanism for anionic surfactants increasing the three-phase contact angle. Sun et al. [32] found that the contact angle of crude oil in anionic surfactants was higher than that in simulated water through macroscopic oil–water–solid contact angle experiments. The results indicated that the surfactant molecules adsorbed at the oil–water interface and solid surface, and generated separation pressure at the three-phase contact point through electrostatic repulsion, which enhanced their ability to strip the oil film on the solid surface.

Moreover, from Table 6 and Table 7, it was observed that both surfactants HABS and PS have the ability to reduce the work of adhesion of the system, and the ability of HABS to reduce the work of adhesion is stronger at higher concentrations. With the gradual increase in the concentration of HABS and PS, the work of adhesion of the system decreases gradually with IFT. Among them, when the three-phase contact angle of HABS is lower, the adhesion work of the system is higher; on the contrary, vice versa. The lower adhesion work indicates the lower separation work required between the crude oil and the solid, and thus the easier the oil film on the solid surface will be stripped.

## 3. Materials and Methods

### 3.1. Materials

Deionized water (prepared in an ion exchange unit), formamide (99% purity), DMF (99% purity), ethanol (99% purity), cyclohexane (99% purity), n-decane (99% purity), n-hexane (99% purity). Surface tension meter (Data Physics, Stuttgart, Germany), Ubbelohde viscometer (SCHOTTAG, Mainz, Germany). Surface free energy of various solvents and their physicochemical properties were cited from the literature [21].

The oil sand particles used in the experiments were supplied by Daqing Oilfield Ltd. (Daqing, China) with a particle size of 147 μm and numbered 1 and 2. The powder samples were sieved using a vibrating sieve (GKM Siebtechnik GmbH, Waibstadt, Germany) to obtain the corresponding particle size. However, it must be noted that the particle size here is an average value due to the dispersion of the powder particles.

The surfactants used in the experiments, HABS (heavy alkyl benzene sulfonate with a molecular weight of about 400) and PS (petroleum sulfonate with a molecular weight of about 400), were supplied by Daqing Oilfield, Heilongjiang. The crude oil was from the Daqing oilfield, with the viscosity of 19.2 cP, the density of 0.84 g/cm^3^, and the surface tension of 29.59 mN/m, at the temperature of the formation of 45 °C. The aqueous phase was simulated water from Daqing formation, prepared with deionized water.

The chemicals CaCl_2_, 6H_2_O-MgCl_2_, Na_2_SO_4_, Na_2_CO_3_, NaHCO_3_ and NaCl used for the preparation of the simulated water were of analytical grade and were purchased from Shanghai McLean Reagent Co. in Shanghai, China. The composition of the simulated water in the Daqing formation is shown in Table 8.

### 3.2. Experimental Methods

#### 3.2.1. Washburn Method

The experimental apparatus was that of the LAUDA Scientific GmbH instrument (LAUDA Scientific GmbH, Stuttgart, Germany), and the experiments were carried out using the Washburn capillary rise method to investigate the wettability of solid particles. Firstly, the sample tube was loaded into the measuring cell, and a piece of filter paper was padded above the exit of the sample tube. A certain mass of powder was weighed and loaded into the glass tube, a weight with a mass of 500 g was hung on the pusher to press the powder, and the height of each impact was kept as consistent as possible [14,33]. Sample loading was completed when the height of the powder bed no longer changed. Operate the inlet pump to add liquid to the measurement cell, the bottom of the powder just in contact with the liquid begins to record the experimental data, until the liquid reaches the top of the powder particles or completely wet the powder, the end of the experiment. Special attention should be paid to the powder bed filling process; each experiment is repeated three times to take the average value.

The Washburn capillary rise method is based on the assumption that powders or porous materials can be described as bundles of capillary tubes with a constant radius [34]. It is derived from the principle of Poiseuille’s law, defined as the existence of a linear relationship between the squared mass, m^2^, and the measurement time, t:

(1) $$m^{2}=\frac{C\rho^{2}\gamma_{\mathrm{LV}}\cos\theta }{\eta }t$$

The above equation can also be reduced to the square of the volume of the solution V^2^ versus the capillary rise time t:

(2) $$V^{2}=\frac{C\gamma_{\mathrm{LV}}\cos\theta }{\eta }t$$

(3) $$C=rA^{2}\varepsilon^{3}$$

Here, V is the volume of the absorbing solution of the sample at a given time (μL), C is the capillary constant of the powder bed (×10^−16^ m^5^), γ_LV_ is the surface tension of the liquid (mN/m), η is the viscosity of the liquid (mPa·s), r is the effective radius of the pores of the powder bed (μm), A is the cross-sectional area of the sample tube (mm^2^), and ε is the void ratio of the powder material. v^2^/t denotes the slope of the wetting curve in the case that there are only two unknowns in the Washburn equation, C and θ. Transform the above equation into the following form:

(4) $$C\times \cos\theta =k\frac{\eta }{\gamma_{\mathrm{LV}}}$$

Here, k represents the wetting slope V^2^/t (μL/s), and the result of multiplying the experimentally measured k with the viscosity η of the solution of the wetted powder and dividing it by the surface tension γ_LV_ gives the result of multiplying the capillary coefficient C with cosθ.

#### 3.2.2. OWRK Method

Owens et al. [35] proposed the OWRK method, where the polarity of a solid can be divided into polar component $\gamma^{p}$ and nonpolar component (dispersion component) $\gamma^{d}$. The relationship between the interfacial free energy $\gamma_{\mathrm{sl}}$ between the solid–liquid two phases and the polar and nonpolar components of the free energy of the solid–liquid surface can be expressed by the OWRK method as follows.

(5) $$\gamma_{\mathrm{sl}}=\gamma_{\mathrm{lv}}+\gamma_{\mathrm{sv}}-2{\left(\gamma_{\mathrm{lv}}^{d}\gamma_{\mathrm{sv}}^{d}\right)}^{1/2}-2{\left(\gamma_{\mathrm{lv}}^{p}\gamma_{\mathrm{sv}}^{p}\right)}^{1/2}$$

In Equation (5): $\gamma_{\mathrm{sv}}^{d}$ and $\gamma_{\mathrm{lv}}^{d}$ are the nonpolar components of the surface free energy of solids and liquids, respectively, and $\gamma_{\mathrm{sv}}^{p}$ and $\gamma_{\mathrm{lv}}^{p}$ are the polar components of the surface free energy of solids and liquids, respectively. Young’s equation is commonly used to calculate the surface free energy of a solid by defining the mechanical equilibrium of a liquid droplet in contact with a solid under three interfacial tensions:

(6) $$\gamma_{\mathrm{sv}}-\gamma_{\mathrm{sl}}=\gamma_{\mathrm{lv}}\cos\theta$$

In Equation (6): $\gamma_{\mathrm{lv}}$, $\gamma_{\mathrm{sv}}$ and $\gamma_{\mathrm{sl}}$ are the surface free energy of the liquid in equilibrium with the saturated vapor of the liquid, the surface free energy of the solid and the interfacial free energy between the solid and the liquid, respectively. Combining Young’s Equation (6) and the OWRK method Equation (5), the joint Equation (7) can be obtained:

(7) $$\frac{\gamma_{\mathrm{lv}}\left(1+\cos\theta \right)}{2\sqrt{\left(\gamma_{l}^{d}\right)}}={\left(\gamma_{s}^{d}\right)}^{\frac{1}{2}}+{\left(\gamma_{s}^{p}\right)}^{\frac{1}{2}}\sqrt{\frac{\gamma_{l}^{p}}{\gamma_{l}^{d}}}$$

In this paper, the powder contact angle θ, the surface free energy of solvent $\gamma_{\mathrm{lv}}$, the polar component $\gamma_{l}^{p}$ and the nonpolar component $\gamma_{l}^{d}$ obtained from Washburn’s method are substituted into Equation (7), and by calculating ${\left(\gamma_{\mathrm{lv}}^{p}/\gamma_{\mathrm{lv}}^{d}\right)}^{1/2}$ of the $\gamma_{\mathrm{lv}}(1+\cos\theta )/2{\left(\gamma_{\mathrm{lv}}^{d}\right)}^{1/2}$ graph, from the slope of the resulting line segment, can be calculated from the polar component of the free energy of the surface of the solid $\gamma_{s}^{p}$, and from the straight line of the intersection of the free energy of the surface of the solid can be calculated from the nonpolar component of the free energy of the solid $\gamma_{s}^{d}$, which are added together to give the surface free energy $\gamma_{s}$ of the solid particle.

#### 3.2.3. Contact Angle Measurements

Contact angle measurements were performed by the fixed-drop method using a LAUDA Scientific OSA 200 device (LAUDA Scientific GmbH, Stuttgart, Germany) [36]. The quartz sheets were cleaned ultrasonically with acetone and ultrapure water for 30 min. Then, the quartz sheets were immersed in chromic acid solution for more than 6 h. The measurements were repeated five times by precipitating droplets (2 μL) on the quartz sheet. The results of five times were averaged, and the standard deviation of the contact angle values was less than 3°.

#### 3.2.4. Surface Tension Measurements

Surface tension measurements were carried out using the Wilhelmy plate method through the surface tension meter (Data Physics, Germany, DCAT21) [37]. Before the start of the experiment, the surface of the platinum plate was heated using an alcohol lamp to remove impurities. The experimental temperature was 45 °C. The accuracy of the measurements was ±0.5 mN/m.

#### 3.2.5. Interfacial Tension Measurements

All IFT data obtained in this paper were obtained by the spin-drop method using a Texas-500C rotating droplet interfacial tensiometer (CNG Enterprises Ltd., Boca Raton, FL, USA). The volume ratio of oil to water was approximately 1:200, the rotational speed input was 5000 r/min, and the experimental temperature was 45 °C. The value of IFT was considered to be at equilibrium when the change in IFT was less than 5% within 60 min. The measurement error of the IFT values was less than ±5% [38].

#### 3.2.6. Adhesion Work

Adhesion work is defined as the work required to separate two dissimilar surfaces. It measures the strength of mutual attraction of surfaces [39]. The work of adhesion of a liquid to a solid surface is derived from the change in free energy of the liquid during wetting of the solid:

(8) $$W_{A}=\gamma_{\mathrm{lv}}+\gamma_{\mathrm{sv}}-\gamma_{\mathrm{sl}}$$

Bring Equation (6) into the above equation:

(9) $$W_{A}=\gamma_{\mathrm{lv}}(1+\cos\theta )$$

In the three-phase contact angle system of oil–water–solid for determining the ability of a solution to strip an oil film from a solid surface, Equation (6) can be expressed as:

(10) $$\gamma_{\mathrm{sl}}-\gamma_{\mathrm{so}}=\gamma_{\mathrm{ol}}\cos\theta$$

In Equation (10): $\gamma_{\mathrm{so}}$, $\gamma_{\mathrm{sl}}$ and $\gamma_{\mathrm{ol}}$ are the interfacial free energies between the solid and the crude oil, between the solid and the solution, and between the crude oil and the solution, respectively, and θ denotes the angle of wetting of the oil droplets in the solution on the solid surface.

For the work of adhesion of the oil phase in solution on a solid surface, it can be expressed by the following equation:

(11) $$W_{A}=\gamma_{\mathrm{ol}}(1+\cos\theta )$$

In Equation (11): W_A_ is the work of adhesion of crude oil on the solid surface in the surfactant solution calculated according to the above equation.

## 4. Conclusions

In this paper, the interaction between anionic surfactant solutions, crude oil and subsurface reservoir rocks was investigated using the Washburn capillary rise method. Based on the experimental results, the following conclusions can be obtained:

(1) For oil sands with low polarity, the wettability of the solvent on the oil sands gradually increases with the decrease in solvent surface tension, and the powder contact angle gradually decreases.

(2) The anionic surfactants HABS and PS adsorbed on the oil sands through hydrophobic interactions. The hydrophilic modification ability was weak for the oil sand surface, and the wetting rate was slightly enhanced with increasing concentration, but the contact angle did not change much in general.

(3) Compared with solvents and surfactants, crude oil has a certain viscosity, and the wetting rate for oil sands is lower. The contact angle of crude oil is close to 90°, resulting in a further decrease in the wetting rate.

(4) The adsorption of HABS and PS on the oil sand surface reduces the solid–liquid interfacial free energy, resulting in higher oil–water–solid three-phase contact angle values for anionic surfactant solutions than for simulated water. At the same time, both HABS and PS have a strong ability to reduce interfacial tension, and the adhesion work is significantly reduced. Therefore, the anionic surfactants have a strong ability to strip the oil film from the solid surface.

## Figures and Tables

**Table 1:** Powder contact angle of different solvents on oil sands 1 and 2.

Contact Angle (θ)	Deionized Water	Formamide	DMF	Cyclohexane	n-C_10_	Ethanol	n-C_6_
Oil sand 1	87.9 ± 0.2	87.7 ± 0.4	84.2 ± 0.5	78.5 ± 0.1	77.5 ± 0.5	77.1 ± 0.2	74.9 ± 0.5
Oil sand 2	88.4 ± 0.2	88.2 ± 0.5	88.4 ± 0.3	77.6 ± 0.1	74.4 ± 0.7	73.8 ± 0.2	71.8 ± 0.4

**Table 2:** Surface free energy of oil sands 1 and 2.

Powder Samples	Particle Size (μm)	$\gamma_{s}^{p}$ (mJ/m^2^)	$\gamma_{s}^{d}$ (mJ/m^2^)	Surface Free Energy (mJ/m^2^)
Oil sand 1	147	9.31	15.21	24.52
Oil sand 2	147	8.82	14.98	23.80

**Table 3:** Powder contact angles of HABS and PS on oil sands 1 and 2, respectively.

HABS	Oil Sand 1	Oil Sand 2	PS	Oil Sand 1	Oil Sand 2
Simulated water	88.6 ± 0.2	88.7 ± 0.5	Simulated water	88.6 ± 0.4	88.7 ± 0.3
1 × 10^−6^	87.1 ± 0.1	87.6 ± 0.2	1 × 10^−7^	88.1 ± 0.1	88.3 ± 0.2
1 × 10^−5^	85.1 ± 0.4	85.4 ± 0.3	1 × 10^−6^	87.6 ± 0.3	87.3 ± 0.2
1 × 10^−4^	85.8 ± 0.1	85.9 ± 0.4	1 × 10^−5^	86.5 ± 0.4	85.9 ± 0.1
5 × 10^−4^	86.2 ± 0.3	86.4 ± 0.1	5 × 10^−5^	85.9 ± 0.3	85.9 ± 0.1
1 × 10^−3^	87.3 ± 0.2	86.9 ± 0.6	1 × 10^−4^	85.8 ± 0.4	86.1 ± 0.2
5 × 10^−3^	87.7 ± 0.2	87.8 ± 0.1	5 × 10^−4^	85.7 ± 0.3	86.1 ± 0.1
1 × 10^−2^	88.1 ± 0.1	88.4 ± 0.1	1 × 10^−3^	85.9 ± 0.4	85.9 ± 0.2

**Table 4:** Interfacial free energies of HABS and PS solutions on oil sands 1 and 2.

HABS (mol/L)	Oil Sand 1 (mJ/m^2^)	Oil Sand 2 (mJ/m^2^)	PS (mol/L)	Oil Sand 1 (mJ/m^2^)	Oil Sand 2 (mJ/m^2^)
Simulated water	22.77	22.23	Simulated water	22.77	22.23
1 × 10^−6^	22.21	21.88	1 × 10^−7^	22.37	21.85
1 × 10^−5^	21.91	21.35	1 × 10^−6^	22.08	21.50
1 × 10^−4^	22.40	21.74	1 × 10^−5^	21.81	21.12
5 × 10^−4^	22.60	22.00	5 × 10^−5^	22.00	21.34
1 × 10^−3^	23.16	22.26	1 × 10^−4^	22.11	21.47
5 × 10^−3^	23.36	22.73	5 × 10^−4^	22.26	21.65
1 × 10^−2^	23.59	22.99	1 × 10^−3^	22.30	21.55

**Table 5:** Free energy of the oil sand–crude oil interface.

Oil Sand	Surface Free Energy of Oil Sand (mJ/m^2^)	C×cosθ (10^−16^ m^5^)	Contact Angle (°)	Free Energy of Solid–Oil (mJ/m^2^)
Oil sand 1	24.52	0.186	88.4 ± 0.2	23.67
Oil sand 2	23.80	0.169	88.5 ± 0.3	23.03

**Table 6:** Oil–water–oil sand 1 three-phase contact angle and adhesion work.

	Concentration	0 wt%	0.005 wt%	0.01 wt%	0.05 wt%
HABS	IFT (mN/m)	13.2	4.1	1.5	0.7
	Contact angle (°)	96.5	108.0	135.4	137.7
	Adhesion work (mJ/m^2^)	13.30	2.83	0.43	0.18
PS	IFT (mN/m)	13.2	5.3	4.5	3.6
	Contact angle (°)	96.5	112.4	108.2	113.1
	Adhesion work (mJ/m^2^)	13.30	3.27	3.09	2.19

**Table 7:** Oil–water–oil sand 2 three-phase contact angle and adhesion work.

	Concentration	0 wt%	0.005 wt%	0.01 wt%	0.05 wt%
HABS	IFT (mN/m)	13.2	4.1	1.5	0.7
	Contact angle (°)	93.9	105.9	117.1	122.7
	Adhesion work (mJ/m^2^)	13.96	2.97	0.81	0.32
PS	IFT (mN/m)	13.2	5.3	4.5	3.6
	Contact angle (°)	93.9	109.0	103.9	105.9
	Adhesion work (mJ/m^2^)	13.96	3.57	3.42	2.61

**Table 8:** Composition of simulated water in the Daqing formation.

Salt	NaCl	KCl	CaCl_2_	MgCl_2_·H_2_O	Na_2_SO_4_	NaHCO_3_	Total
Concentration (mg/L)	2294	13	42	172	75	1860	4456

## Data Availability

Data contained within the article.
